# Effect of Intercritical Tempering Temperature on Microstructure Evolution and Mechanical Properties of High Strength and Toughness Medium Manganese Steel

**DOI:** 10.3390/ma15062162

**Published:** 2022-03-15

**Authors:** Xiaokai Liang, Hang Fu, Mei Cui, Gang Liu

**Affiliations:** 1 Department of Structural Steels, Central Iron and Steel Research Institute Company Limited, Beijing 100081, China; liangxk@sina.com (X.L.); cuimei@cisri.com.cn (M.C.); 2 NCS Testing Technology Co., Ltd., Beijing 100081, China; fh626983712@126.com; 3 Beijing Advanced Innovation Center for Materials Genome Engineering, University of Science and Technology Beijing, Beijing 100083, China

**Keywords:** medium manganese steel, intercritical tempering temperature, reversed austenite, mechanical properties, microstructure evolution

## Abstract

The effect of intercritical tempering temperature (TT) on the microstructure evolution and mechanical properties of 3.6Mn medium manganese steel, which contained martensite and austenite, was investigated by X-ray diffraction, electron backscattering diffraction and transmission electron microscopy, as well as Thermo-Calc calculation. The results showed that the volume fraction of reversed austenite (RA) increased firstly and then decreased with the increasing TT in the range of 550~650 °C. When the TT was below 620 °C, lath-like RA with good stability was mainly displayed between martensite laths and its size is about 100 nm. When the TT was higher than 650 °C, larger-size and block RA was formed in the martensite block boundaries, and part of the RA transformed into fresh martensite during cooling. The yield strength and tensile strength of the experimental steels decreased gradually as the TT increased, but the tensile strength increased gradually with the formation of block RA and fresh martensite. Lath-like RA could significantly improve the toughness and plasticity with slight loss of yield strength, but block RA decreased slightly them.

## 1. Introduction

It is well known that the advanced high strength steel (AHSS) strongly depends on the content of Mn, which is regard as an alternative element to Ni to reduce the costs [1]. Ni significantly improves the low-temperature toughness in 9Ni steel; however, the yield strength is usually at the low level so that the application is limited [2]. As the third generation AHSS, medium manganese steels with the content of Mn varying from 3.0 to 12.0 (wt.%) possess excellent comprehensive mechanical properties, such as high strength and low-temperature toughness, which have been applied to exploitation equipment of oil and gas, automobile industry, building trades, and so on [3,4]. A large number of studies have shown that the tensile properties can be obtained by introducing a certain volume fraction of dispersed and stable reversed austenite (RA) in materials containing a multiphase microstructure [5,6]. However, the effect of RA on high yield strength and good toughness is worth being further investigated.

Stabilized by enough Mn and C, RA remains at room temperature, or it would transform into fresh martensite during cooling [7]. In addition, a proper amount of RA transforms into martensite when subjected to deformation, namely a transformation-induced plasticity (TRIP) effect, which delays the formation of necking and greatly improves plasticity [8]. The TRIP effect also significantly improves the low-temperature toughness through a crack tip passivation effect, local transformation induced plasticity, and purification of the matrix [9]. According to this potential toughening and plasticizing mechanism, the development of medium manganese steel gains excellent comprehensive mechanical properties [10,11,12]. However, as the soft phase, the yield strength decreases with an increase in RA content [5]. Therefore, the proper heat treatment process used to control the microstructure and mechanical properties has become a research hotspot [13,14].

The RA is usually introduced by the vital intercritical heat treatment process in α + γ two phase regions, where both Mn and C, as the austenite stabilization elements, diffuse into the substructure boundaries for austenite nucleation [15]. The heat treatment parameters, such as the intercritical tempering time and the intercritical tempering temperature (TT), directly affect the content and morphology of RA, and also determines the mechanical properties [16]. In this paper, the medium manganese martensite and austenite multiphase steel is designed as the research object, using austenite homogenization quenching and an intercritical tempering heat treatment process to produce RA, and using X-ray diffraction (XRD) technology to detect the RA content. Observe the microstructure evolution after tempered at different temperatures by electron backscattering diffraction (EBSD) and transmission electron microscopy (TEM). The mechanical properties were tested, and the effect law of TT on microstructure and mechanical properties is studied and discussed for guiding the design of a new type structural steel.

## 2. Materials and Methods

The experimental steel was melted in a 200 kg vacuum induction furnace, and hot-forged into a round bar with a size of Φ 20 × 950 mm^2^ between 900 °C and 1100 °C. The nominal composition (wt.%) was listed in Table 1. Thermo-Calc software, TCFE 10.0, was used to calculate the equilibrium phase transformation temperature points: A_e1_ = 592 °C and A_e3_ = 732 °C (where A_e1_ is the starting temperature of α-phase to γ-phase transformation, and A_e3_ is the temperature at which all α-phase is transformed into γ-phase). Considering the above phase transformation characterizations, the steel was preserved at a temperature of 780 °C for 40 min and then was oil-cooled to room temperature. Subsequently, the steel was tempered at 550, 580, 600, 620 and 650 °C, respectively. The tempering time was 3 h, and the steel was cooled to room temperature in oil.

**Figure 1:**
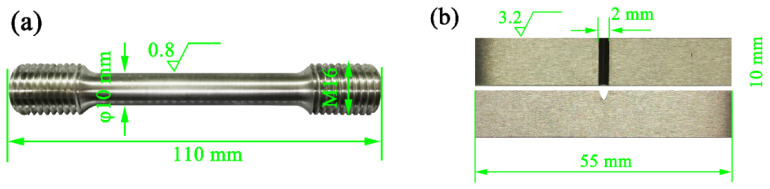
Mechanical test samples pictures: (**a**) Tensile tensile tests; (**b**) impact tensile tests.

The room temperature tensile tests of the tempered steels were performed in a WE-300 hydraulic tensile testing machine (Jinan Chenxin testing machine Manufacturing Co., LTD., Jinan, China). The standard tensile specimen was machined with a diameter of 5 mm along the forging direction by the GB/T228.1-2010. The Charpy impact tests with V notch basing on the GB/T 229-2020 was tested in a JBN-300B testing machine (Jinan Hengda Huifeng Test Instrument Co., LTD., Jinan, China), and the test temperature was 0 °C and −40 °C, respectively and the mechanical test samples pictures were shown in Figure 1. The tensile properties were determined by two values, and the impact energy was averaged from three values. The impact fracture morphology and microstructure were observed by the FEI Quanta 650 scanning electron microscopy (SEM) (FEI Company, Hillsboro, OR, USA).

The SEM and EBSD samples were 5 mm thick, 10 mm long and 10 mm wide. After grinding and polishing, the samples were electrolytically polished in a mixed solution of 6% perchloric acid ethyl alcohol. The Oxford Nordly F+ electron backscatter diffraction (EBSD) (Oxford Instruments, Abingdon, England) was used to observe the microstructure, and the scanning step was 0.2 μm. Slices were cut from the tempered steel and mechanically ground to a thickness of about 50 μm. The Gatan 656 pit machine (Gatan, Pleasanton, CA, USA) was used to obtain several discs with a diameter of 3 mm. Next, the discs were thinned by the Tenupol-5 double electrolytic jet (Struuers, Copenhagen, Denmark) in the 10% perchloric acid ethyl alcohol solution. Subsequently, the samples were observed in an H-800 transmission electron microscope (TEM) (HITACHI, Tokyo, Japan). PANALYTICAL-MPD X-ray diffractometer (XRD) (PANalytical, Almelo, The Netherlands) was used to measure the RA volume fraction of the tempered steel at room temperature in accordance with GB 8362-87 (step size of 0.02°, and step scan range of 45~115°). The specific calculation formula was following [17]:

(1) $$V_{\gamma }=\frac{I_{\gamma }}{I_{\gamma }+1.4I_{\alpha }}$$

where *V*_γ_ is the volume fraction of RA. *I*_α_ and *I*_γ_ are the cumulative intensity of the diffraction peaks of ferrite and austenite crystal planes, respectively.

## 3. Results

### 3.1. Mechanical Properties

**Figure 2:**
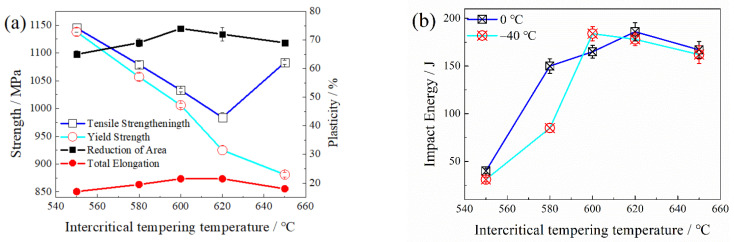
Mechanical properties after tempered at different temperatures: (**a**) strength and plasticity and (**b**) impact energy.

Figure 2 shows the tensile properties at room temperature, and the impact energies at 0 °C and −40 °C of the tempered steel. With an increase of TT, the yield strength gradually decreased from 1138 MPa (550 °C) to 681MPa (650 °C). The tensile strength first decreased and then increased, and the minimum value was 984 MPa at 620 °C and increased to 1100 MPa at 650 °C. The yield ratio was significantly reduced with the increase of TT. The elongation and reduction of area of the tempered steel reached their peaks at 600 °C, which were 21.5% and 74%, respectively.

Figure 2b shows the impact energy tested at 0 °C and −40 °C, respectively. When tempered in the single α phase region (below A_e1_), the impact energy was 40 J (tested at 0 °C) and 31 J (tested at −40 °C). As the TT was above A_e1_ temperature, the impact energy was significantly improved. The maximum impact energy tested at 0 °C was 186 J when the steel was tempered at 620 °C, while the maximum impact energy tested at −40 °C was 184 J when the steel was tempered at 600 °C. As the TT increased to 650 °C, the impact energy decreased slightly.

### 3.2. Fracture Morphology

**Figure 3:**
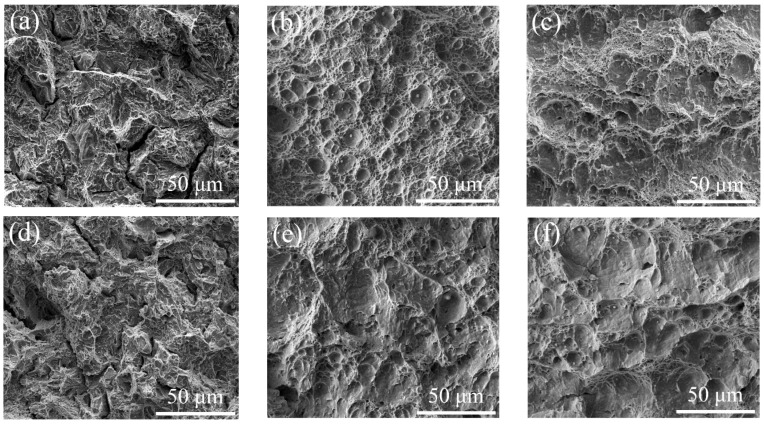
Fracture morphology of the tempered steel: (**a**) 550 °C, tested at 0 °C; (**b**) 620 °C, tested at 0 °C; (**c**) 650 °C, tested at 0 °C; (**d**) 550 °C, tested at −40 °C; (**e**) 600 °C, tested at −40 °C; (**f**) 650 °C, tested at −40 °C.

Figure 3 shows the fracture morphology of the experimental steel after it was subjected to tempering at different temperatures. After being tempered at 550 °C, there was a quasi-cleavage fracture mode. The unit of quasi-cleavage fracture got smaller when the test temperature decreased from 0 °C to −40 °C (Figure 3a,d). Shallow dimple fracture mode appeared when the highest impact energy was acquired (Figure 3b,e). When the TT increased to 650 °C, the counts of dimple decreased and the depth of dimple became shallower so that some dimples were hard to follow.

### 3.3. Microstructure Characterization

#### 3.3.1. SEM Characterization

**Figure 4:**
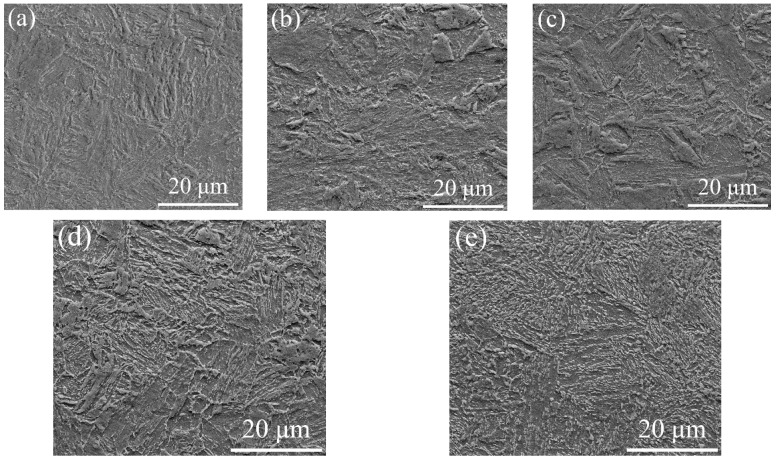
SEM images: (**a**) 550 °C; (**b**) 580 °C; (**c**) 600 °C; (**d**) 620 °C; (**e**) 650 °C.

Figure 4 displays the microstructure evolution as the TT increased. After being subjected to tempering, the characteristics of some martensite laths disappeared. With an increase in TT, the martensite substructure boundaries coarsened, and some block microstructure appeared. The portion of block microstructure increased to about 30% when tempered at 650 °C. It was hard to distinguish the microstructure from each other, and the microstructure was more homogeneous (Figure 4e).

#### 3.3.2. XRD Characterization of RA

**Figure 5:**
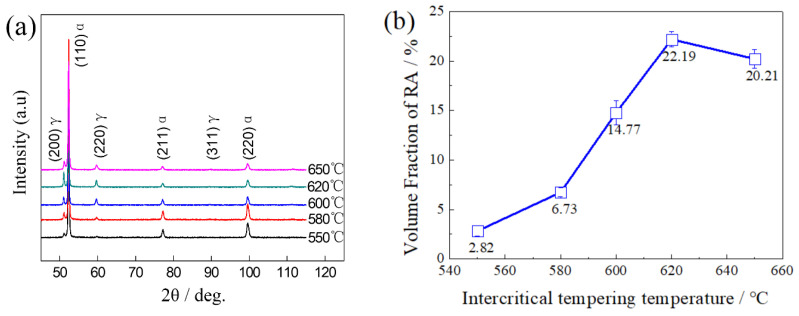
(**a**) XRD line spectra patterns, and (**b**) the tested volume fraction of RA.

The diffraction peaks of RA of tempered steels were detected by XRD, and the volume fraction was calculated using Equation (1). The XRD line spectrum patterns were shown in Figure 5a, where (111)_γ_, (200)_γ_, (220)_γ_, (311)_γ_ were the diffraction peaks of RA, and the rest were diffraction peaks of α-Fe [18,19]. It could be seen that the diffraction peak intensity of RA was strongest when the TT was 620 °C, representing the highest volume fraction of RA. Figure 5b shows the volume fraction of RA after being tempered at different temperatures. It could be seen that as the TT increased, the volume fraction of RA first increased and then decreased, reaching the maximum value of 22.19% at 620 °C.

#### 3.3.3. EBSD Characterization of Effective Grains

**Figure 6:**
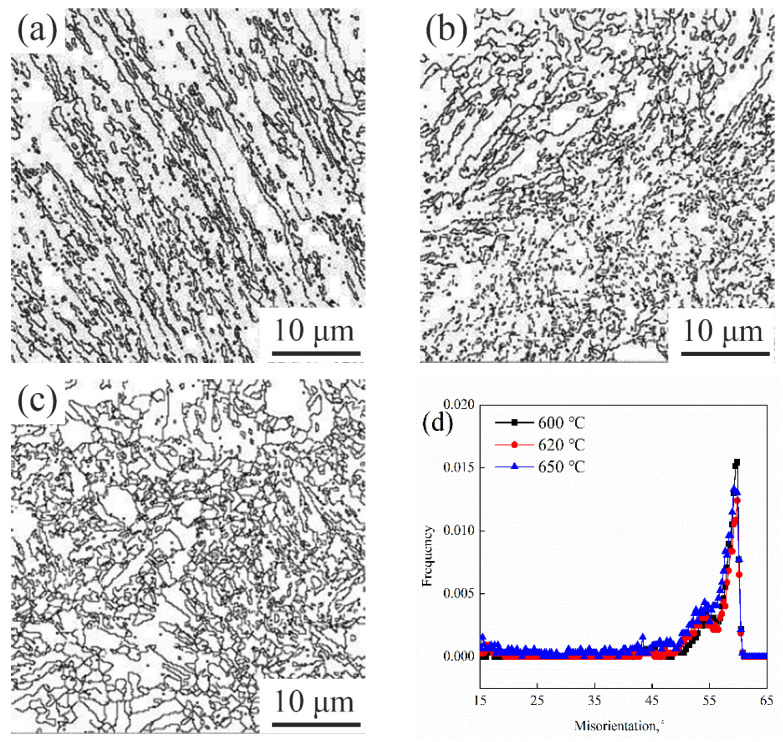
Boundary distribution maps with misorientation angle beyond 15° and misorientation angle distribution maps: (**a**) 600 °C; (**b**) 620 °C; (**c**) 650 °C; (**d**) misorientation angle distribution maps.

The so-called effective grains referred to grains surrounded by high angle grain boundaries, where the misorientation angle difference was above 15 degrees [20]. The EBSD was used to detect the effective grain size, and the grain boundary distribution was showed in Figure 6. It could be seen that the effective grain size tempered between 600 °C and 620 °C was significantly smaller than that tempered at 650 °C.

#### 3.3.4. EBSD Characterization of RA

**Figure 7:**
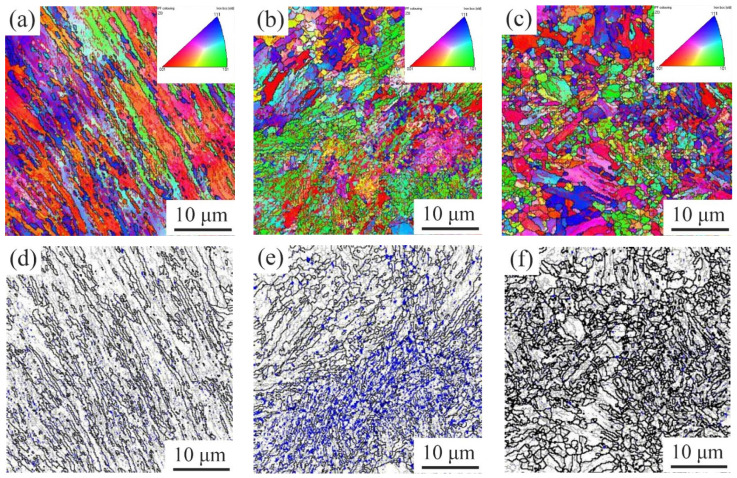
EBSD analysis of RA: (**a**,**d**) 600 °C; (**b**,**e**) 620 °C; (**c**,**f**) 650 °C.

Figure 7 showed the EBSD characterization results of the experimental steel. Figure 7a,c,e were the IPF images, and Figure 6b,d,f were the superposition of both grain boundaries and phases. The white area represented the α phase and the blue area represented the γ phase. It could be seen that with an increase of TT, the characteristics of martensite lath gradually disappeared, and an obvious block to RA appeared after it was tempered at 620 °C, which was mainly dispersed in the high angle trigeminal grain boundary and the low angle grain boundary, and also displayed between the martensite laths. The average size of RA was about 1 μm. As the TT further increased, the amount of block RA decreased instead.

The EBSD RA content in the tempered steels was 1.95% at 600 °C, 13.4% at 620 °C, and 1.36% at 650 °C, which were significantly lower than the tested results by XRD. One of the reasons was the limitation of the EBSD scanning step size (0.2 μm) so that the smaller austenite, less than 200 nm, could not be detected [19]. The other was that the larger austenite transformed into fresh martensite due to the poor thermal or mechanical stability [19].

#### 3.3.5. TEM Characterization of RA

**Figure 8:**
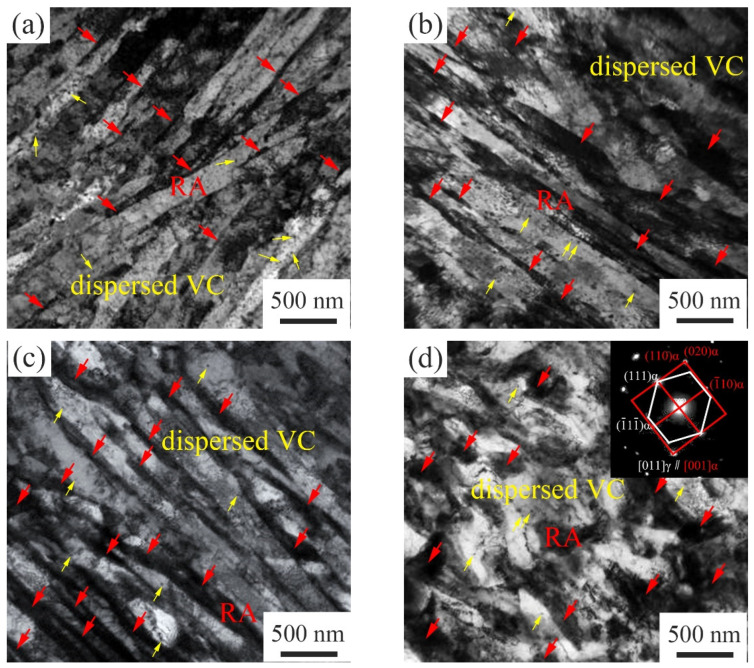
TEM images and the selected area electron diffraction pattern: (**a**) 600 °C; (**b**) 620 °C; (**c**,**d**) 650 °C.

Figure 8 shows the TEM morphology and the selected area electron diffraction pattern of RA after being tempered at different temperatures. The lath-like RA was mainly displayed between the tempered martensite laths, being parallel to the martensite lath, and the size was about 100 nm. When the TT was 650 °C, a large amount of block RA was found (Figure 8d), which was consistent with the EBSD observation. There was a typical Kurdjumov–Sachs (K–S) crystallographic orientation relationship between RA and tempered martensite [21]: (111)_α_//(110)_γ_; [001]_α_//(110)_γ_ (Figure 8d).

## 4. Discussion

### 4.1. Effect of TT on RA Stability

**Figure 9:**
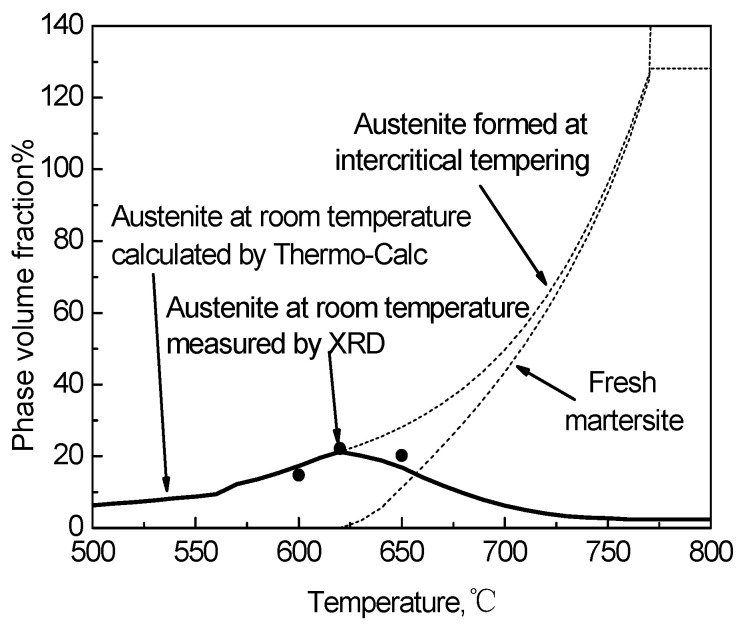
Phase volume fraction calculated by Thermo-Calc and measured by XRD.

When the TT is higher than the A_e1_ temperature, the austenite phase is gradually formed. Due to the different solid solubility of alloying elements in tempered martensite and RA, elements such as C and Mn gradually diffuse to RA so that the stability of the RA is significantly increased [19]. RA can still be preserved at room temperature after subjected to oil cooling. Thermo-Calc software can be used to calculate the mass fraction of each phase during tempering at different temperatures and the mass fraction of alloying elements such as carbon and manganese in the RA in the equilibrium state [22,23]. Substituting the calculated mass fraction of the alloying elements in the austenite into the empirical Formula (2), the martensite starting temperature Ms of RA can be calculated [24]:

Ms = 532 − 391.2[C] − 43.3[Mn] − 21.8[Ni] − 16.2[Cr](2)

where, [M] is the mass fraction (%) of alloying elements in RA in the equilibrium state calculated according to Thermo-Calc. Then the Koistinen–Marburger equation can be used to calculate the volume fraction of RA transformed into fresh martensite [25]:

f_M_ = 1 − e^−0.011(Ms−T)^ (3)

where, Ms is the martensite starting temperature of RA; T is room temperature; f_M_ is the volume fraction of fresh martensite generated when cooled below Ms; the volume fraction of RA at room temperature was equal to the difference of austenite volume fraction in equilibrium and the volume fraction of fresh martensite. Figure 9 shows the volume fractions of the various phases in the tempered steel at different temperatures are obtained through the above model of both calculations and XRD measurements.

**Figure 10:**
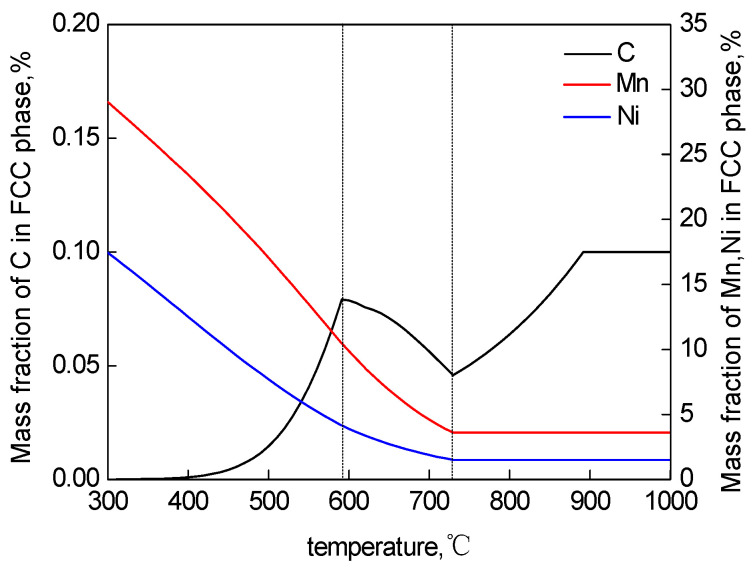
Mass fraction of C, Mn and Ni in γ at different temperatures calculated by Thermo-Calc.

The RA content calculated by the model is basically consistent with the XRD measured value, which can be used to estimate the relationship between the TT and the RA content, being conducive to the optimization of the heat treatment process [26]. When the TT is lower than 620 °C, almost all of the RA remains at room temperature, forming metastable retained austenite. In this temperature range, the higher the content of alloying elements in RA is, and the higher the stability is. When the TT is higher than 620 °C, part of the RA transforms into fresh martensite during oil cooling, which reduces the RA content. Figure 10 shows that Thermo-Calc calculates the changes in the mass fraction of each component in the austenite phase varying with temperature in the equilibrium state. As the TT increases, the content of austenite stabilizing elements containing carbon, manganese and nickel in the austenite phase gradually decreases so that the chemical stability decreases [27].

With an increase of TT, the proportion of austenite gradually increases (Figure 9), but the content of Mn and Ni gradually decreases (Figure 10). Until the TT is higher than A_e3_, the matrix is completely transformed into the austenite phase, and the content of manganese and nickel reaches an equilibrium state. With an increase of the TT, the C mass fraction in the austenite phase has three inflection points. The first point at 600 °C is mainly due to the dissolution of the cementite phase, the second point of C mass fraction where the ferrite phase is completely transformed into the austenite phase is of a minimal value, and the third point shows that the VC phase is completely dissolved in austenite. Before the first point, the variation in the C mass fraction in the austenite phase is mainly affected by the cementite phase [28]. Between the first point and the second point, the C mass fraction primarily varies with the increase in austenite content. As the temperature increases from the second point to the third point, the solid solubility of the VC phase in the austenite phase gradually decreases and the carbon content in the austenite phase begins to increase again until the VC phase is completely dissolved in austenite.

When the TT is lower than 620 °C, the volume fraction of RA is low, but the average concentration of the content of austenite stabilizing elements C, Mn and Ni is higher. Meanwhile, no martensite transformation occurs during oil cooling so that almost all RA remains at room temperature. The RA is mainly formed between the tempered martensite laths in lath-like, and the arrangement direction is parallel to the surrounding martensite laths, which increases the resistance to the transformation from austenite to martensite. When the TT exceeds 620 °C, although the content of the RA increases, the austenite stabilizing elements C, Mn and Ni content are lower, and the size is larger, together leading to a decrease in the RA stability [19]. A large amount of block RA is formed at the intersection of the martensite boundaries. When the block RA undergoes martensitic transformation, the expansion restraint force is small, which further reduces the stability of the austenite for forming fresh martensite. The martensite transformation results in a gradual decrease in the RA content at room temperature. When the TT is 620 °C, the experimental steel has the highest RA content.

### 4.2. Effect of TT on Mechanical Properties

The multi-phase structure has different effects on the tensile strength and yield strength of experimental steel. The decisive factor for the tensile strength is the volume fraction of the hard phase in the matrix, while the austenite in the steel is a soft phase with a low volume fraction [29], and martensite is a hard phase. In the two-phase region tempering, the tensile strength of the experimental steel first decreases and then increases with an increase of TT, which was mainly because the RA partially formed transformed into fresh martensite. As the TT increases, the α phase gradually softens, resulting in a gradual decrease in the yield strength.

The effect of the multiphase structure on the plasticity is mainly due to the following two aspects: on the one hand, as the proportion of the austenite phase in the multiphase structure increases, the plasticity of the steel increases; on the other hand, the Transformation Induced Plasticity (TRIP) effect will be induced during deformation [30]. When part of the RA during oil cooling is transformed into fresh martensite, the plasticity of the steel is also reduced. When the TT is 620 °C, the elongation and reduction of the area reaches the maximum.

When the TT was below A_e1_, the quasi-cleavage fracture mode is observed (Figure 3a,d), because the content of RA is so low that toughness is not significantly improved (Figure 5b). When subjected to intercritical tempering, lath-like RA plays an important role in improving the toughness: (1) In the process of crack propagation, the crack tip is passivated, the stress concentration is weakened, and the crack propagation is hindered [31,32]. (2) The RA formed at the grain boundary position can dissolve C, Mn and other elements, so that the grain boundary is purified, and the grain boundary strength is significantly improved [33,34]. (3) During the fracture process, there is a large stress concentration at the crack tip. When the crack tip encounters RA, a TRIP effect occurs under the action of the stress field, which absorbs energy and relieves the stress concentration at the crack tip, hinders the propagation of the crack, and improves the low temperature toughness of the steel [33,34,35]. The reduction of effective grain size increases the area of the boundary that can hinder crack propagation, reduces the concentration of impurity elements on the grain boundary to avoid brittle fracture along the crystal, improves the impact energy of the steel on the platform and reduces the ductile-brittle transition temperature. When the steel is tempered at 620 °C, the amount of RA reaches its peak, and it mainly exists in the lath-like form. The RA has a high stability and the impact energy of the steel also reaches its peak, leading to the appearance of a dimple fracture mode. As the TT continues to increase, the volume fraction and stability of RA in the steel will decrease, and the proportion of block RA will increase. The block RA is conducive to improving the plasticity of the steel. However, at a lower plastic deformation and less energy, less martensite transformation occurs, and the effect of hindering crack propagation is not obvious, and the effect of improving low-temperature impact toughness is significantly reduced [36]. Therefore, the counts of dimple decreases and the depth of dimple becomes shallower.

## 5. Conclusions

1. As the TT increases, the volume fraction of RA first increases and then decreases. When the TT is lower than 620 °C, the RA with a high stability is mainly in lath-like form displayed between martensite laths. When the TT is higher than 620 °C, the content of block RA gradually increases, and the content of stable elements such as C, Mn and Ni in RA decreases. Part of the RA transforms into fresh martensite during oil cooling, resulting in a decrease in the RA content.
2. The yield strength and tensile strength gradually decrease with the increase of the TT, but the tensile strength gradually increases in view of the formation of block austenite and fresh martensite.
3. Lath-like RA can significantly improve the toughness and plasticity. With the formation of block RA, the toughness and plasticity decrease slightly. When tempered at 620 °C, the plasticity and toughness reach a maximum. The fracture mode of low-temperature impact changes from a quasi-cleavage fracture mode to dimple fracture mode with the increasing content of RA.

## Figures and Tables

**Table 1:** Chemical composition of experimental steel.

C	Si	Mn	P	S	V + Cr + Mo + Ni + Cu	Fe
0.10	0.10	3.60	0.008	0.005	4.33	Bal.

## Data Availability

The data presented in this study are available on request from the corresponding author.
